# Mutations in *Escherichia coli aceE* and *ribB* Genes Allow Survival of Strains Defective in the First Step of the Isoprenoid Biosynthesis Pathway

**DOI:** 10.1371/journal.pone.0043775

**Published:** 2012-08-21

**Authors:** Jordi Perez-Gil, Eva Maria Uros, Susanna Sauret-Güeto, L. Maria Lois, James Kirby, Minobu Nishimoto, Edward E. K. Baidoo, Jay D. Keasling, Albert Boronat, Manuel Rodriguez-Concepcion

**Affiliations:** 1 Centre for Research in Agricultural Genomics (CRAG) CSIC-IRTA-UAB-UB, Campus UAB Bellaterra, Barcelona, Spain; 2 Joint BioEnergy Institute, Emeryville, California, United States of America; 3 Department de Bioquímica i Biologia Molecular, Universitat de Barcelona, Barcelona, Spain; University Of Montana – Missoula, United States of America

## Abstract

A functional 2-*C*-methyl-D-erythritol 4-phosphate (MEP) pathway is required for isoprenoid biosynthesis and hence survival in *Escherichia coli* and most other bacteria. In the first two steps of the pathway, MEP is produced from the central metabolic intermediates pyruvate and glyceraldehyde 3-phosphate *via* 1-deoxy-D-xylulose 5-phosphate (DXP) by the activity of the enzymes DXP synthase (DXS) and DXP reductoisomerase (DXR). Because the MEP pathway is absent from humans, it was proposed as a promising new target to develop new antibiotics. However, the lethal phenotype caused by the deletion of DXS or DXR was found to be suppressed with a relatively high efficiency by unidentified mutations. Here we report that several mutations in the unrelated genes *aceE* and *ribB* rescue growth of DXS-defective mutants because the encoded enzymes allowed the production of sufficient DXP *in vivo*. Together, this work unveils the diversity of mechanisms that can evolve in bacteria to circumvent a blockage of the first step of the MEP pathway.

## Introduction

Isoprenoids (also called terpenoids) are a ubiquitous and highly diverse family of compounds produced from the five-carbon precursor isopentenyl diphosphate (IPP) and its double-bond isomer dimethylallyl diphosphate (DMAPP) in all living organisms [1]–[3]. These precursors are synthesized from acetyl-CoA by the mevalonic acid (MVA) pathway in archaea (archaebacteria), fungi, and animals. By contrast, the unrelated 2-*C*-methyl-D-erythritol 4-phosphate (MEP) pathway produces IPP and DMAPP from pyruvate and glyceraldehyde 3-phosphate in most bacteria (eubacteria) and apicomplexan protozoa, including important human pathogens such as those causing tuberculosis and malaria [3]–[5]. Because the MEP pathway is essential in such pathogens but is not present in animals, it has been proposed as a promising target for the design of new antibacterial and antimalarial agents that would be potentially innocuous for humans [6]–[8]. However, we still know little about possible mechanisms of resistance to current and potential drugs targeting the MEP pathway in bacterial pathogens.

**Figure 1:**
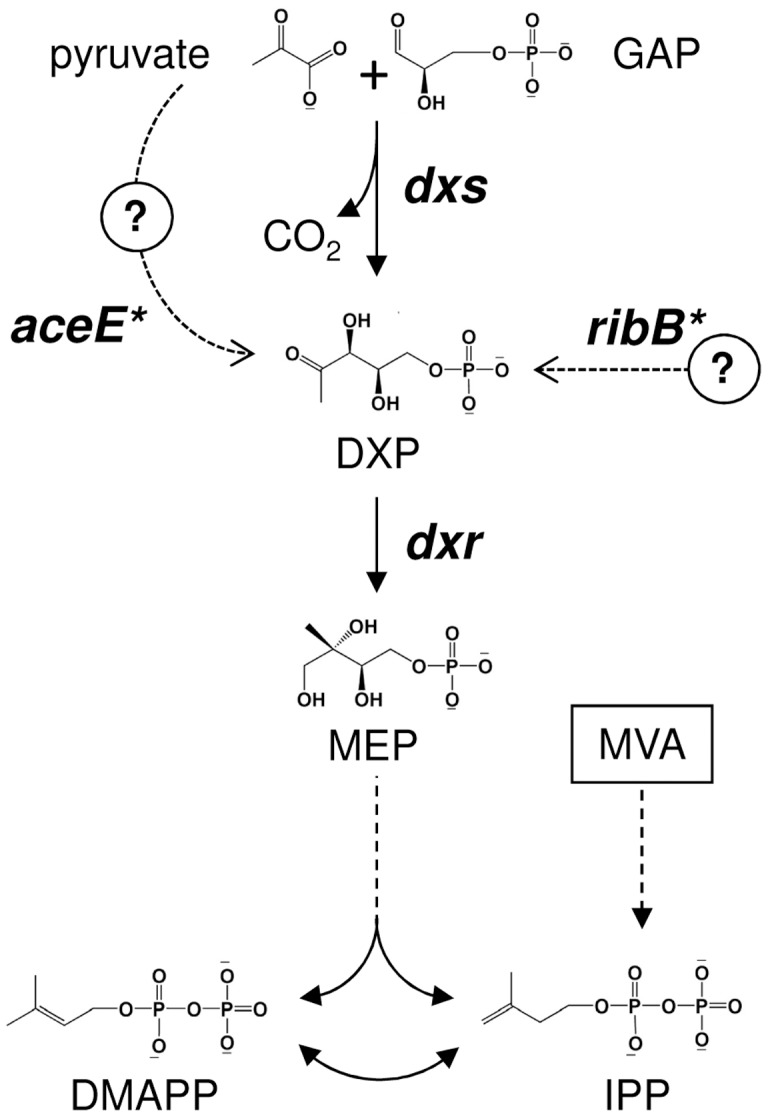
Biosynthesis of isoprenoid precursors in *E. coli*. The indicated genes (in italics) encode enzymes that produce the first intermediates of the MEP pathway either originally (*dxs*, *dxr*) or by mutation as indicated by asterisks (*aceE*, *ribB*). The *E. coli* strains used in this work are engineered to synthesize isopentenyl diphosphate (IPP) and dimethylallyl diphosphate (DMAPP) from exogenously supplied mevalonic acid (MVA). GAP, D-glyceraldehyde 3-phosphate; DXP, 1-deoxy-D-xylulose 5-phosphate; MEP, 2-*C*-methyl-D-erythritol 4-phosphate.

The genes and enzymes of the MEP pathway are best characterized in *Escherichia coli*. The initial reaction of the pathway (Fig. 1) is catalyzed by 1-deoxy-D-xylulose 5-phosphate (DXP) synthase (DXS, encoded by the *dxs* gene) and involves the condensation of (hydroxyethyl)thiamine, derived from pyruvate, with the C1 aldehyde group of D-glyceraldehyde 3-phosphate to produce DXP [9]–[11]. In the second step, an intramolecular rearrangement and reduction of DXP by the enzyme DXP reductoisomerase (DXR, encoded by the *ispC/yaeE/dxr* gene) yields MEP [12], [13]. An alternative oxidoreductase enzyme with a DXR-like (DRL) activity was recently found in a reduced number of bacteria [14]. MEP produced by DXR or DRL is eventually converted to both IPP and DMAPP by sequential activities of the enzymes encoded by the genes *ispD/ygbP*, *ispE/ychB*, *ispF/ygbP*, *ispG/gcpE*, and *ispH/lytB* [5], [15].

The best studied inhibitor of the MEP pathway is fosmidomycin (FSM), a specific inhibitor of the enzyme DXR which also inhibits DRL [14], [16]–[19]. The uptake of FSM by bacterial cells is an active process involving a cAMP-dependent glycerol 3-phosphate transporter (GlpT) protein [20]. Loss of GlpT activity in *E. coli* mutants or the absence of a GlpT homologue in other bacteria such as *Mycobacterium tuberculosis* or *Brucella abortus* leads to FSM resistance [14], [20], [21]. Enhanced export of the inhibitor by overexpression of the *E. coli fsr* gene also results in FSM resistance [22]. Antibiotic resistance can result not only from interfering with drug transport or mode of action but also from the use of an alternative pathway not affected by the inhibitor. To investigate the relevance of the latter type of mechanisms for resistance to MEP pathway inhibitors, we aimed to identify spontaneous mutations that could suppress an otherwise lethal obstruction of the pathway in living bacteria.

Loss of function of the MEP pathway in *E. coli* can be rescued in strains containing a synthetic MVA operon that allows the production of IPP and DMAPP from exogenously supplied MVA (Fig. 1) [23], [24]. However, in cells harboring a deletion of the *dxs* gene (strain EcAB4-2), MVA auxotrophy was suppressed with a relatively high frequency (6.4 per 10^9^ cells) by mutations in other genes [25]. As a result, colonies of DXS-defective suppressor mutants could grow overnight on plates lacking MVA. Suppressor mutants were also found in the DXR-deficient strain EcAB4-10, although with a slightly lower frequency (2.4 per 10^9^ cells) and poor growth. No suppressor mutants were found in strains with disruptions to the other MEP pathway genes [25]. These results suggested that bacteria can respond to a block of DXS or DXR activities by using other proteins that deliver DXP or MEP when mutated [25]. In this work we identify genes and mutations that allow survival of DXS-deficient strains and demonstrate that the mutant proteins are indeed able to synthesize DXP (or a precursor molecule) *in vivo*.

## Materials and Methods

### Bacterial strains and screening for suppressor mutants

Disruption of individual MEP pathway genes in *E. coli* strains EcAB4-2 (*dxs::CAT*) and EcAB4-10 (*dxr::CAT*), each containing a chromosomal copy of a synthetic MVA operon, was carried out as described [25] by deleting most of their coding region and introducing chloramphenicol resistance (*CAT*) genes. Similarly, strain EcAB1-6 (*dxs::CAT dxr::TET*) was constructed by disrupting both *dxs* and *dxr* genes with chloramphenicol and tetracycline resistance genes, respectively, in MC4100 cells harboring the MVA operon in plasmid pAB-M3 [23], [26]. For isolation of suppressor mutants, EcAB4-2 cells were cultured at 37°C in Luria broth (LB) medium supplemented with 1 mM MVA, 25 µg/ml kanamycin (to select for the MVA operon) and 17 µg/ml chloramphenicol (to select for the disruption of the *dxs* gene) until exponential phase. After cells were pelleted and rinsed twice with LB, several batches of *ca*.10^8^ cells (estimated by measuring optical density at 600 nm, OD600) were plated on LB agar containing only kanamycin and chloramphenicol. In some plates, a paper disk soaked in 30 µl ethylmethane sulphonate (EMS) was placed on the surface of the medium to induce mutations. Spontaneous and EMS-induced mutants that formed a colony in the absence of MVA were analyzed to confirm the deletion of the *dxs* genes by PCR.

### Identification of the genes mutated in the suppressor lines

Genomic DNA from suppressor mutants was isolated as described [25], [27] and partially digested with *Sau*3A. Fragments of 2–4 kb were gel-purified using the Qiaquick (Qiagen) system and ligated into the *Bam*HI site of pUC19. After amplification of the genomic library in TOP10 cells, EcAB4-2 competent cells were transformed with aliquots of the library, and transformants resistant to kanamycin, chloramphenicol, and 100 µg/ml ampicillin that were able to grow without MVA were selected. Plasmid DNA isolated from these transformants was used to transform new EcAB4-2 cells to confirm that it conferred the ability to grow in the absence of MVA. Positive clones were sequenced to identify the gene (and the mutation) responsible for the suppression phenotype. Screenings for mutations specifically in *aceE* and *ribB* were carried out by amplification of their coding regions by colony PCR with high-fidelity AccuPrime DNA polymerase (Invitrogen), followed by sequencing.

### Plasmid constructs

The wild-type and mutant *aceE* and *ribB* genes (including the promoter region) were amplified from EcAB4-2 and suppressor mutant cells by PCR using AccuPrime DNA polymerase and primers aceE-1F (5′- C C A G A A G A T G T T G T A A A T C A A G C -3′) and aceE-4R (5′- T T T A C C T C T T A C G C C A G A C G -3′) for *aceE* and ribB-pNF (5′- A G C A T A T G A G T G C C A T T G T A G T G-3′) and ribB-XR (5′- A G T C A C T C G A G G C T G G C T T T A C G C T C A T G T G C-3′) for *ribB*. The PCR products were gel-purified and cloned into pCRII-TOPO (Invitrogen). Inserts were sequenced using vector and gene-specific primers to confirm the presence of the identified mutations.

### Detection of DXP in cell extracts

EcAB1-6 cells were transformed with plasmids harboring wild type and mutant versions of *aceE* or *ribB* (or an empty pCRII-TOPO plasmid as a control) and positive transformants were selected on LB plates supplemented with 1 mM MVA, 50 µg/ml kanamycin (to select for the pCRII-TOPO constructs), 100 µg/ml ampicillin (to select for the pAB-M3 plasmid with the MVA operon), 17 µg/ml chloramphenicol (to select for the disruption of the *dxs* gene), and 5 µg/ml tetracyclin (to select for the disruption of the *dxr* gene). Individual colonies were then grown at 37°C overnight in liquid media with the same supplements. The overnight cultures were used to confirm the identity of the strain and plasmids by PCR and restriction analysis and to inoculate fresh 10 ml cultures to an OD600 of 0.05. Inoculated cultures were grown at 37°C for 5 hours and aliquots corresponding to an OD600 of 10 were then collected. Cells were pelleted and immediately resuspended in 200 μl of 1∶1 (v/v) methanol: water. Cell suspensions were loaded in Amicon Ultra-0.5 centrifugal filter devices and spun at 14,000 rpm and 4°C for 90 min in a refrigerated microfuge. DXP was detected and quantified in the flow-through by liquid chromatography and mass spectrometry (LC-MS).

LC was conducted on an ZIC-pHILIC column of 150-mm length, 2.1-mm internal diameter, and 5-µm particle size (Merck SeQuant), using an Agilent 1200 Series HPLC system. A sample injection volume of 2 µL was used throughout. The temperature of the sample tray was maintained at 4°C by an Agilent FC/ALS Thermostat. The column compartment was set to 50°C. The mobile phases used were composed of A) 80 mM ammonium carbonate and B) acetonitrile. Isocratic elution was achieved at 68% B via a flow rate of 0.23 mL/min for 12 min. From there the flow rate was gradually increased to 0.3 mL/min until 12.5 min. The flow rate was then held at 0.3 mL/min for a further 2.5 min. The HPLC system was coupled to an Agilent 6210 time-of-flight mass spectrometer (LC-TOF MS), by a 1/3 post-column split. Contact between both instrument set-ups was established by a LAN card in order to trigger the MS into operation upon the initiation of a run cycle from the MassHunter workstation (Agilent). Nitrogen gas was used as both the nebulizing and drying gases to facilitate the production of gas-phase ions. The drying and nebulizing gases were set to 10 L/min and 25 psi, respectively, and a drying gas temperature of 300°C was used throughout. Electrospray ionization (ESI) was conducted in the negative ion mode and a capillary voltage of –3500 V was utilized. MS experiments were carried out in the full scan mode, at 0.86 spectra/ sec and a cycle time of 1.1162.8 sec, for the detection of [M–H]- ions. The instrument was tuned for a range of 50–1700 m/z. Prior to LC-ESI-TOF MS analysis, the TOF MS was calibrated via an ESI-L-low concentration tuning mix (Agilent). Data acquisition and processing were performed by the MassHunter software package. DXP was quantified via a five-point calibration curve. The R2 coefficient for the calibration curve was >0.99. The chemical standard for DXP (Echelon) was made up to 500 µM, as the stock solution, in methanol-water (50∶50, v/v).

## Results

### Most DXS-defective suppressor mutants harbour mutations in the aceE gene

To investigate the mechanisms by which bacteria could survive in the absence of a DXS enzyme, we aimed to identify the genes responsible in a collection of DXS-defective suppressor mutants (SX lines). Previous screening for suppressors of MVA auxotrophy in DXS-defective EcAB4-2 cells (i.e. the lethality caused by *dxs* deletion) resulted in the isolation of 6 spontaneous mutants (SX1 to SX6) [25]. A second screening led to the isolation of 14 more spontaneous mutants (SX7 to SX20) and 14 EMS-induced mutants (SX1E to SX14E). However, 6 spontaneous mutants and 2 EMS-induced mutants lost the ability to grow without MVA after storage as glycerol stocks and re-streaking on fresh LB plates supplemented only with kanamycin and ampicillin. Of the remaining 26 mutants, one (SX5) was previously reported to harbor a missense mutation in the *aceE* gene (encoding the catalytic E1 subunit of the pyruvate dehydrogenase complex, PDH), resulting in an E636Q change in the protein sequence [25]. Sequencing of the *aceE* gene in the remaining mutants identified a total of 4 different mutations (Table 1). In particular, the E636Q mutation was found in 2 other strains besides SX5, whereas 1 clone had a different mutation in the same residue (E636G). Two more *aceE* mutations were found: Q408R in 1 strain and L633R in 17 clones. In total, 22 out of 26 suppressor SX mutants contained mutations in the *aceE* gene, suggesting that the alteration of PDH activity might be the most common mechanism of resistance to the inhibition of DXS activity.

**Table 1:** Ocurrence of identified mutations in EcAB4-2 cells.

	Mutation		Ocurrence
Gene	DNA	Protein	(# strains)
*aceE*	cag > cgg	Q408R	1
	ctg > cgg	L633R	17
	gaa > caa	E636Q	3
	gaa > gga	E636G	1
*ribB*	ggt > agt	G108S	2
	gac > ggc	D113G	2

### A mutant ribB gene was found in the remainder of the DXS-deficient suppressors

To identify the genetic change responsible for the bypass of DXS activity in the 4 remaining strains with no mutations in *aceE* (SX6, SX7, SX18, and SX12E), we followed a strategy similar to that previously described for SX5 [25]. We selected strain SX18 to construct a genomic library in pUC19. After amplification, DXS-defective EcAB4-2 cells were transformed with the library, and colonies able to grow without MVA were selected to isolate the incorporated plasmid and sequence the corresponding inserts. All the isolated plasmids were found to contain a mutation in the *ribB* gene, encoding 3,4-dihydroxy-2-butanone 4-phosphate synthase (DHBPS). This enzyme catalyzes the conversion of D-ribulose 5-phosphate to formate and 3,4-dihydroxy-2-butanone 4-phosphate, the latter serving as the biosynthetic precursor for the xylene ring of riboflavin [28]. Complete sequencing of the *ribB* gene in strains SX6, SX7 and SX12E also found mutations in this gene. In particular, a missense mutation resulting in a G108S change in DHBPS was found in SX18 and SX12E, whereas clones SX6 and SX7 harbored a D113G change (Table 1).

### The identified mutations are sufficient to rescue the loss of DXS activity in EcAB4-2 cells

**Figure 2:**
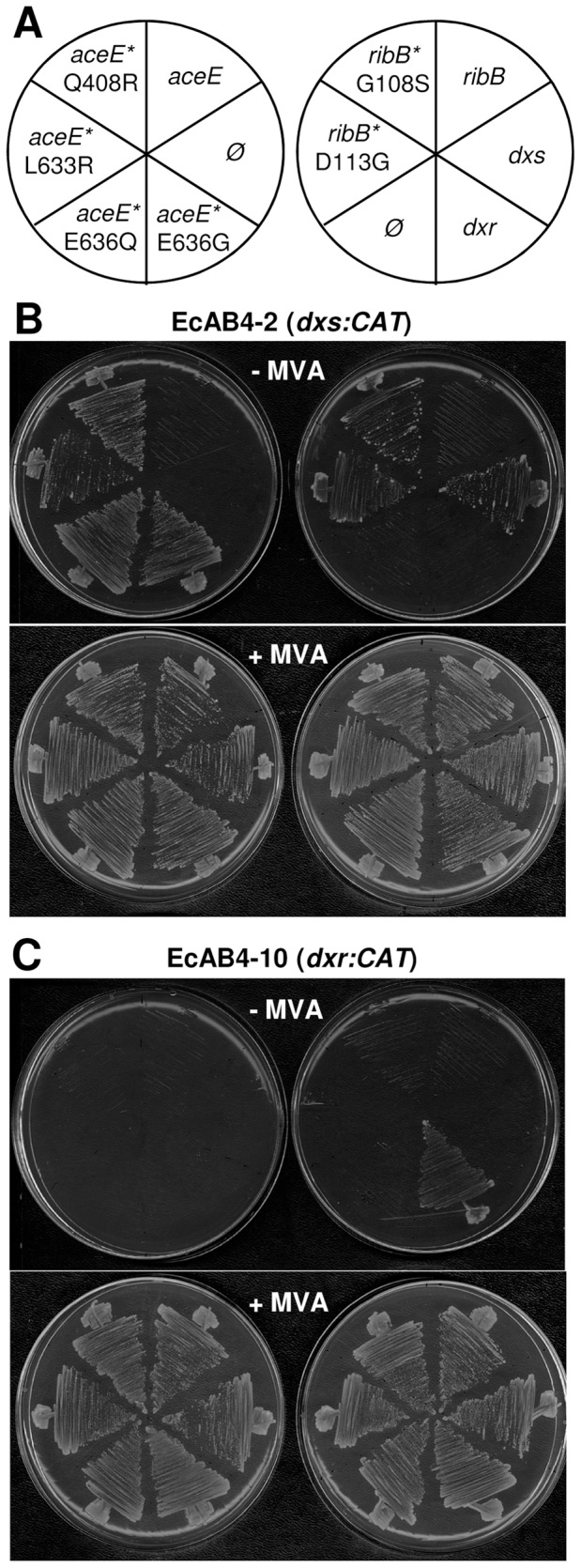
Complementation of *E.coli* strains defective in DXS or DXR. Cells were transformed with pCRII-TOPO constructs and plated on LB medium containing chloramphenicol (for the disruption of chromosomal *dxs* or *dxr* genes), kanamycin (for the MVA operon) and ampicillin (for the introduced plasmid). The medium was supplemented (+) or not (−) with MVA as indicated. Plates were incubated at 37°C for 20 h. (A) Position of transformants harboring plasmids with the indicated wild type or mutant (asterisks) genes or an empty vector control (Ø). (B) EcAB4-2 (*dxs::CAT*) transformants. (C) EcAB4-10 (*dxr::CAT*) transformants.

To confirm whether the new mutations identified in *aceE* and *ribB* were actually responsible for the suppression phenotype, wild type and mutant versions of these genes (including their promoters) were amplified from the parental EcAB4-2 strain and the corresponding suppressor mutants, respectively, and used to complement EcAB4-2 cells. As shown previously [25], the plasmid carrying the E636Q mutation in *aceE* rescued growth on plates without MVA, whereas the wild type gene did not complement the loss of DXS activity in EcAB4-2 (Fig. 2). The other *aceE* mutations (Q408R, L633R, and E636G) were also able to rescue MVA auxotrophy of the strain. Similarly, the two identified mutant forms of *ribB* (G108S and D113G), but not the wild type version, rescued growth of DXS-defective EcAB4-2 cells in medium without MVA (Fig. 2). These results suggest that the presence of any of the identified mutations in *aceE* or *ribB* results in altered enzymes (PDH or DHBPS, respectively) that each produce a metabolite that can ultimately be converted to isoprenoids in the absence of a functional DXS activity.

### Mutant aceE and ribB genes allow the production of DXP in vivo

The bypass of DXS by mutant forms of PDH and DHBPS enzymes might result either from the production of DXP or an alternative substrate for DXR, or from the supply of MEP or a downstream metabolite of the pathway (Fig. 1). The latter possibility can be addressed by using a genetic strategy based on the complementation of the DXR-deficient strain EcAB4-10. Thus, the putative production of MEP or a downstream MEP pathway intermediate by the mutant enzymes would allow not only DXS-defective but also DXR-defective cells to grow in the absence of MVA (Fig. 1). However, when EcAB4-10 cells were transformed with the constructs containing mutant *aceE* and *ribB* genes, transformants could only be recovered on MVA-containing plates because none of them were able to rescue MVA auxotrophy (Fig. 2). These results confirm that the mutant PDH and DHBPS enzymes synthesize a product that enters the MEP pathway upstream of the reaction catalyzed by DXR.

**Figure 3:**
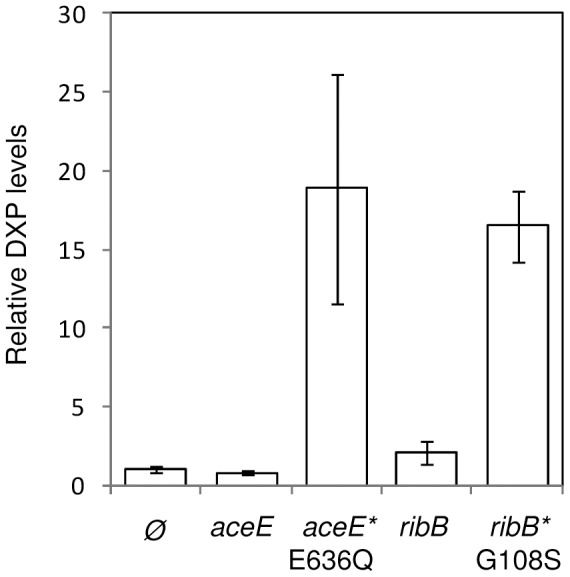
DXP levels in cells expressing wild type and mutant *aceE* and *ribB* genes. *E.coli* cells defective in both DXS and DXR were transformed with pCRII-TOPO constructs with the indicated wild type or mutant genes or an empty vector control (Ø). Positive transformants were grown in triplicate for 5 h with MVA, and DXP levels were measured by LC-MS. Mean and standard deviation (n = 3) values are represented relative to the levels in Ø controls.

To discriminate between the remaining possibilities (i.e. the mutant enzymes allow the production of either DXP or another metabolite that can be used as an alternative substrate for DXR), we investigated whether the presence of mutant versions of PDH or DHBPS resulted in the production of DXP in cells lacking both DXS and DXR activities (strain EcAB1-6). After transformation of the EcAB1-6 strain with plasmids containing wild type and mutant versions of both *aceE* and *ribB*, cells were grown in MVA-supplemented liquid medium and harvested in the exponential growth phase. HPLC-MS/MS analysis of cell extracts from controls transformed with an empty plasmid showed the presence of low but detectable levels of DXP (Fig. 3), indicating that *E. coli* cells devoid of DXS activity can still produce trace amounts of this metabolite. Levels of DXP in transformants carrying plasmids with wild type versions of *aceE* or *ribB* genes were similar to those in the negative control. By contrast, strains expressing the mutant genes showed *ca*. 20-fold higher DXP levels (Fig. 3), confirming that the corresponding mutant enzymes are able to synthesize DXP (or a metabolite that can be transformed into DXP by the cells) *in vivo*.

## Discussion

In bacteria, isoprenoids play essential roles in a variety of processes that are vital for growth and survival, including cell wall and membrane biosynthesis, electron transport, and conversion of light into chemical energy [29]. Also, many microorganisms produce isoprenoid secondary metabolites of economic relevance [30]. Since the discovery of the MEP pathway in the mid 1990 s, it became more and more evident that bacteria display a wide metabolic plasticity regarding the route to produce isoprenoids. Most bacteria only use the MEP pathway for IPP and DMAPP biosynthesis, but there are exceptions to this trend. Some bacteria synthesize their isoprenoid precursors using the MVA pathway instead of the MEP pathway, whereas there are parasitic bacteria that lack both pathways (likely because they obtain their isoprenoids from host cells) and also organisms that possess the two full pathways [3], [31]–[34]. Most strikingly, related bacteria may use different pathways for isoprenoid biosynthesis while unrelated bacteria may use the same pathway. In addition to the MEP and MVA pathways, alternative pathways and metabolic intermediates have been proposed for isoprenoid biosynthesis in the cyanobacterium *Synechocystis* PCC 6803, which lacks the MVA pathway and does not appear to use the canonical MEP pathway under photosynthetic conditions [35], [36]. Diversity can be found also at the level of individual pathway steps, with several examples of reactions catalyzed by different classes of enzymes with no sequence, structural, or catalytic similarity [14], [17], [33], [37]. In at least some cases, it is clear that the alternative routes to MEP pathway intermediates resulted from the recruitment of available enzymes that were not related to isoprenoid synthesis. But we still know very little about the genes and enzymes that could potentially contribute to isoprenoid biosynthesis, despite the relevance of this knowledge to both development of efficient antibiotics and metabolic engineering of isoprenoid biosynthesis. Here we show that the MEP pathway intermediate DXP can be produced (although at very low levels) in the absence of a functional DXS-encoding gene, suggesting that other enzymes might produce this metabolite, perhaps as a by-product. However, such trace levels are not sufficient to support bacterial growth and survival. We also provide biochemical and genetic evidence of novel routes for efficient DXP synthesis arising from specific mutations in two *E. coli* genes, *aceE* and *ribB* (encoding PDH and DHBPS, respectively).

The E636Q mutation in PDH was previously shown to rescue the deficiency of DXS but not DXR activity *in vivo* [25]. In this work, we have identified three more mutations (Q408R, L633R, and E636G) that allow PDH to catalyze an unknown reaction that eventually results in the production of DXP in the absence of endogenous DXS activity. Like DXS, PDH is a thiamine diphosphate (TPP) dependent carboligase that catalyzes the decarboxylation of pyruvate with the formation of hydroxyethyl-TPP as an intermediate. In fact, purified PDH has been shown to catalyze the formation of 1-deoxy-D-xylulose (DX) from pyruvate and D-glyceraldehyde [38], [39]. DX can be phosphorylated to DXP by the kinase encoded by the *xylB* gene in *E. coli* [40]. However, our results suggest that *in vivo* the wild type PDH enzyme does not produce enough DX (or DXP, using D-glyceraldehyde 3-phosphate) to rescue the loss of DXS activity since only the mutant enzyme is able to complement the *dxs* knockout (Fig. 2) and to cause an accumulation of DXP beyond background levels (Fig. 3).

In the case of DHBPS, the proposed mechanism for the conversion of D-ribulose 5-phosphate to formate and 3,4-dihydroxy-2-butanone 4-phosphate involves a complex series of steps including dehydration, intramolecular rearrangement and rehydration [41]. Interestingly, the alternative route into the MEP pathway proposed in *Synechocystis* PCC 6803 appears to be stimulated by D-xylulose 5-phosphate and also by dihydroxyacetone phosphate [35], [36]. We therefore speculate that either D-ribulose 5-phosphate or its isomer D-xylulose 5-phospate might be converted to DXP by mutant G108S or D113G enzymes. We intend to further investigate the metabolic route to the DXP pathway enabled by mutant DHBPS enzymes using these and alternative substrates and considering the option that a metabolite may be generated that serves as an intermediate for DXP synthesis.

In summary, our work demonstrates that bacteria can circumvent a blockage of the MEP pathway at the level of DXS by mutation of either of two genes (*aceE* and *ribB*). Based on these results, DXS is not a good target for the development of new antibiotics, since resistance is likely to be developed very easily. On the other hand, DXP production appears to be one of the main flux-determining steps of the MEP pathway because overexpression of DXS-encoding genes has been shown to result in an enhanced production of MEP-derived isoprenoids in bacteria and plants [30], [42]. We are currently investigating whether the use of mutant PDH and DHBPS enzymes could be a good strategy to overproduce DXP in metabolic engineering approaches.
